# The impact of Covid-19 restrictions on depressive symptoms in low-risk and high-risk pregnant women: a cross-sectional study before and during pandemic

**DOI:** 10.1186/s12884-022-04515-3

**Published:** 2022-03-08

**Authors:** Martina Smorti, Angelo Gemignani, Lucia Bonassi, Giulia Mauri, Alessia Carducci, Chiara Ionio

**Affiliations:** 1 Department of Surgical, Medical and Molecular Pathology and Critical Care Medicine, University of Pisa, Via Savi 10, 56126 Pisa, Italy; 2 Department of Mental Health, ASST Bergamo-Est, Seriate, Italy; 3 Catholic University of Milano, Milano, Italy

**Keywords:** Low-risk pregnancy, High-risk pregnancy, Hospitalization, COVID-19 restrictions, Depressive symptoms, Open-ended question

## Abstract

**Background:**

The COVID-19 social restrictions have increased the risk for depression compared to the previous period in Italian women with Low-Risk Pregnancy (LRP). lLess is known about the impact of COVID-19 restrictions on High-Risk Pregnancy (HRP). This study aimed: 1) to explore levels of depression in women who become pregnant before and during COVID-19 pandemic, distinguishing between LRP and HRP; 2) to analyze the impact of COVID-19 restrictions on pregnancy experience in LRP and HRP.

**Methods:**

A before-during COVID-19 pandemic cross-sectional study was carried out on 155 pregnant women (Mean age = 34.18), between 23 and 32 weeks of gestation. 77 women were recruited before COVID-19 pandemic (51.9% LRP; 48.1% HRP) and 78 women were recruited during COVID-19 pandemic (51.3% LRP; 48.7% HRP). HRP group was enrolled during hospitalization for high-risk pregnancy. Participants filled out Edinburgh Postnatal Depression Scale. Moreover, only COVID-19 group answered an open-ended question about the impact of restriction on pregnancy experience.

**Results:**

HRP women reported higher levels of depressive symptoms than LRP. No difference emerged for COVID (before/during) but an interaction effect between COVID-19 and obstetric condition was found. The qualitative results showed the impact of restrictions on emotions and concerns.

**Conclusion:**

Respect to the previous period, LRP women during COVID-19 presented an increased risk for depressive symptoms than HRP. The HRP women during COVID-19 seemed to use hospitalization as a resource to find a social support network with other pregnant women and to be reassured on the clinical ongoing of pregnancy.

## Background

Pregnancy constitutes a significant period in a woman’s life where specific emotional needs increase the vulnerability of mental health. The COVID-19 pandemic and the consequent social restrictions have increased the risk of perinatal depression compared to the previous period [1–4].

Analyzing how the COVID-19 pandemic and restrictions have changed the emotional experience of pregnancy has become relevant especially in Italy. This was the first European Country to adopt national lockdown to limit the spread of the disease and, after the first, a second lockdown has been adopted. Physical distancing rules were imposed during the period of March–May and October–December 2020, with the closure of all nonessential services. Additional measures including working, and studying from home were adopted by government to assist in physical distancing: social occasions were banned, and severe restrictions on individual movement were adopted [5] (DPCM 24/10/2020; DPCM 03/12/2020). These public health policies profoundly disrupted normal social interactions removing the chance to gather with close ones and limiting the moments of social exchange and sharing. Consequently an increase in depressive symptoms in the general population has been registered [6]. Overall, the restrictive physical and social distancing measures were the strictest and longest in Europe, at least in 2020 (BBC https://www.bbc.com/news/av/world-europe-52400085).

In addition to general restriction policy, Italian hospitals also changed policies on perinatal care (i.e. deleting nonessential visits, and barring visitors such as partners from the delivery room and postpartum units) in order to achieve a higher security for both mother and child. Despite the higher security for maternal/foetal physical health, these restrictive policy led Italian women who become pregnant during COVID-19 to increase the risk for perinatal depression compared to pregnant women before the pandemic [7, 8].

Qualitative investigations during the COVID-19 pandemic showed that pregnant women expressed feelings of fear, sadness and loneliness in response to their gestation and childbirth possibly due to lack of partner. These emotions seem related to the perception of childbirth, and maternity services modifications induced by pandemic restriction [9].

Despite a large consensus exists about the negative impact of restriction policies on perinatal psychological wellbeing, most of studies focused on women with healthy pregnancy (or Low-Risk Pregnancy -LRP) [1, 2, 4] neglecting women with High-Risk Pregnancy (HRP). If it would emerge an increased depression in women with HRP during COVID-19 pandemic (respect to the pre-COVID-19), clinical implication would be relevant. In fact, literature displayed that depressive symptoms during pregnancy are associated with preterm delivery [10] and that preterm delivery is associated with several adverse neonatal outcomes (admission to neonatal intensive care unit, neurological disability, and mortality) [11].

Studies that referred to pre-COVID-19 pandemic showed that the level of depressive symptoms in women with HRP is higher than that reported by LRP women [12, 13]. In fact, given that the low risk pregnancy is generally managed in a not-hospitalized setting while the high-risk pregnancy is often hospitalized, hospitalization might constitute an additional risk factor for women’s mental health [12–14]. The separation from partner, and the perception of being alone in the management of emergency procedures were the main worries reported by women hospitalized due to HRP in the pre-COVID-19; these worries increased the depressive symptoms [15].

Given the strict restrictive measure adopted in sanitary setting during COVID-19 pandemic, it is reasonable to suppose that women with HRP during the pandemic, experience a greater isolation from their partner and significant others than those with HRP in the previous period. Therefore, the isolation and lack of concrete social exchange and sharing with close ones experienced by HRP women during COVID-19 might increase the risk of depressive symptoms in this population respect to previous period. However, to our knowledge there are no Italian studies that compare the depressive symptoms in women with HRP before and during the pandemic.

This paper has a two-fold purpose. Firstly, to investigate the impact of both the obstetric condition (LRP versus HRP) and the COVID-19 condition (pre- and during- pandemic restrictions) on the level of depressive symptoms in pregnant women. Secondly, to analyze the impact of COVID-19 restrictions on experience of pregnancy in LRP and HRP. Specifically, the main interest is to focus on the emotions and thoughts related to pregnancy.

## Methods

### Procedure and participants

To these aims, a pre- and during COVID-19 pandemic cross-sectional study was conducted using quantitative and qualitative method. This study was conducted following the Strengthening the Reporting of Observational studies in Epidemiology (STROBE) guidelines. All experiments were performed in accordance with relevant guidelines and regulations.

This study was part of a larger research project on the psychological wellbeing of pregnant women out in three-level unit of a maternity ward in University Hospital in Tuscany, Italy since 2018 (Institute Ethics Committee approval n.12749/2018). Subsequently the outbreak of the COVID-19, we adapted the data collection to investigate the impact of COVID-19 restrictions.

For this study the sample was recruited between October 2019 and December 2020 by responsible for the study. The sample was composed by 155 pregnant women aged between 21 and 47. The sample was divided according to COVID-19 condition (77 before and 78 during pandemic restrictions) and to pregnancy obstetric condition (80 women with LRP and 75 with HRP). Specifically, pregnant women during the COVID-19 (LRP and HRP) were recruited during pandemic strict isolation period, between October and December 2020. Pregnant women before the COVID-19 pandemic (LRP and HRP) were recruited in the same Hospital between October and December 2019.

According to obstetric condition, women with LRP were recruited during routine visit for third trimester in the University Hospital in Tuscany according to follow inclusion criteria: aged > 18 and < 39 years old, Italian nationality, low-risk pregnancy which involve no evidence of any of the following conditions: hypertension, pre-eclampsia, gestational diabetes, congenital anomalies, premature rupture of membranes, symptoms of premature birth, intrauterine growth restriction [16], without any experience of hospitalization during pregnancy.

The psychologist responsible for the study approached women waiting for routine obstetric visit in the Gynaecology and Obstetrics ward of the Hospital and, after informing them about the study, she asked for voluntary participation.

Women with HRP were recruited according to specific inclusion criteria, indicated by medical staff, regarding the risk conditions for premature delivery associated to low infant birth weight, and other poor outcomes [17]. These criteria were: aged > 18 years old, Italian nationality, hospitalized, under 33 weeks of gestation, with conditions of risk for preterm delivery (such as premature rupture of membranes, intrauterine growth restriction, symptoms of premature birth, twin pregnancy at risk). The cut-off of 33 weeks gestation was chosen because infants born before 33 weeks, defined “very preterm”, are at higher risk for perinatal mortality, and neonatal complications [18]. In order to prevent a possible preterm labor, the HRP woman needed to be hospitalized for > 4 days for monitoring their obstetric condition. The psychologist responsible for the study approached women while they were hospitalized in the Gynaecology and Obstetrics ward of the Hospital and, after informing them about the study, she asked for voluntary participation. Questionnaires were administered during the hospitalization. HRP group during COVID-19 pandemic were enrolled in COVID-19 free ward.

Women who agreed to participate signing the written consent filled out a battery of questionnaires to register their socio-demographical data and the level of depression. Moreover, only COVID-19 group was requested to answer to an open ended additional question about the impact of COVID-19 restrictions on pregnancy experience in terms of daily life changes, emotions and thoughts.

Only completed questionnaires were accepted for this study.

### Instruments

Obstetric and clinical data (gestational age, presence of assisted reproductive technology) were extracted by clinical record.

The battery of questionnaire was aimed to assess:

- *Socio-demographical data*, including age, educational level, marital status, parity, model of conception, previous miscarriages.
- *Prenatal Depression*. The Italian version of the *Edinburgh Postnatal Depression Scale* (EPDS) also validated against women during pregnancy was used to assess the presence of depressive symptoms [19–21]. Respondents are required to indicate, for each of 10 items, how often they experienced depressive symptoms by rating them on a four-point scale (from 0 to 3). EPDS total score ranges from 0 to 30, with higher scores corresponding to a higher severity of depressive symptoms. In accordance with epidemiological studies, we used the cut-off EPDS score ≥ 10 as a measure for prenatal depression. In the present study, the Cronbach’s alpha coefficient was .88.
- *Open ended question.* Only women recruited during COVID-19 pandemic were asked to respond to one open-ended additional question. This question aimed to collect data about the impact of COVID-19 and consequent restrictions on the emotional experience of pregnancy during the last 2 weeks and the strategies used to cope with emotions. In particular, the question had a neutral formulation to permit women to express both positive and negative feelings related to the experience of pregnancy during COVID-19. Women were asked: “How do the pandemic restrictions impact on your experience of pregnancy in the last two weeks? Please report your thoughts, emotions, feelings, behaviours”

### Data analysis

A post hoc power analysis was conducted using the software package, GPower 3.1.9.4. This post hoc analyses revealed the statistical power for this study was .73.

Quantitative data were analyzed using the Statistical Package for Social Sciences (SPSS), version 24 (2017). Comparisons among the four groups in sociodemographic, clinical and obstetrics data were performed with one way ANOVA. Qualitative variables were summarized as counts and percentages and comparisons among groups were evaluated with Chi square test.

To explore the role of the COVID-19 condition and gestational obstetric condition on the level of depression (assessed through EPDS), a univariate analyses of covariance (ANCOVAs), with the COVID-19 group (before and during pandemic) and obstetric condition (LRP vs HRP) as fixed factors, the dimension of the EPDS as dependent variable and the variables significantly different among groups (use of assisted reproductive technologies) as covariate. Finally, to explore the distribution of participants positive and negative to depression in four groups, we conducted a Chi square test with four groups (pre-COVID-19 LRP, pre-COVID-19 HRP, during COVID-19 LRP, during COVID-19 HRP) and clinical value of EPDS as dependent variable.

For multiple comparisons, Bonferroni corrections were applied. The alpha level was set to *p* = .05 for all tests with confidence interval at 95%.

Qualitative data (answers to open-ended questions) were analyzed by two independent coders who read the transcript to identify themes, patterns, salient points [22, 23]. The two coders classified the content together, working to come to an agreement when their evaluations differed.

## Results

The sample was composed by 155 pregnant women divided in four groups. Table 1 reported the characteristics of the study sample.

**Table 1:** Characteristics of study sample

	Pre- COVID-19		COVID-19		Total sample *N* = 155
	LRP *N* = 40	HRP *N* = 37	LRP *N* = 40	HRP *N* = 38	
Age range (years)	27–39	23–47	21–37	21–46	21–47
Age, Median (years)	34.5	35	34	33.6	34
Weeks of gestation^a^, range	25–32	23–32	27–32	23–32	23–32
Week of gestation, median	29	30	30	30	30

*LRP* Low-Risk Pregnancy, *HRP* High-Risk Pregnancy

^a^when included in the study

### Comparison of demographic and obstetric characteristics

Table 2 showed the demographic, obstetric characteristics of sample.

**Table 2:** Comparison between group for demographic and obstetric data

	Pre- COVID-19				COVID-19			
	LRP		HRP		LRP		HRP	
	N	%	N	%	N	%	N	%
Age years, mean (SD)	35.17 (3.74)		34.51 (6.21)		33.45 (4.42)		33.61 (5.44)	
Weeks of gestation, mean (SD)	29.2 (2.14)		28.8 (3.04)		30.00 (1.32)		29.76 (2.15)	
Scholarship Years, mean (SD)	16.50 (3.96)		15.62 (4.39)		16.78 (2.19)		15.24 (4.46)	
Couple relationship								
Married/cohabitant	39	97.5	36	97.3	40	100	38	100
In couple but not cohabitant	1	2.5	1	2.7	0	–	0	–
Pregnancy								
Single	40	100	34	91.9	40	100	35	92.2
Twin			3	8.1			3	7.8
Mode of conception								
Spontaneous	36	90	27	73	38	95	29	76.3
Assisted reproductive treatment	4	10	10	27	2	5	9	23.7
Parity								
Primiparous	23	57.5	28	75.7	32	80	28	73.7
Multiparous	17	42.5	9	24.3	8	20	10	26.3
Previous miscarriage								
No	34	85	32	86.5	38	95	28	73.7
Yes	6	15	5	13.5	2	5	10	26.3
*Reason of hospitalization, n (%)*								
Premature Rupture Of Membranes			6	16.2			3	7.9
Risk of preterm delivery			20	54.1			18	47.4
Fetal growth restriction			5	13.5			5	13.2
Other			6	16.2			12	31.6

*LRP* Low-Risk Pregnancy, *HRP* High-Risk Pregnancy

No significant difference was found among the four group with respect to mean age (F_(3,151)_ = 1.03; *p* = .38), mean gestational age (F_(3,151)_ = 10.61; *p* = .10), mean educational years level (F_(3,151)_ = 1.38; *p* = .25), marital status (χ^2^_(1)_ = 1.12; *p* = .29), Single pregnancy (χ^2^_(3)_ = 6.60; *p* = .08), previous miscarriage (χ^2^_(3)_ = 3.13; *p* = .37), parity (χ^2^_(3)_ = 5.68; *p* = .13). On the contrary, the four groups differ for mode of conception (χ^2^_(3)_ = 9.63; *p* = .02).

Moreover, no difference exits for reason of hospitalization within HRP group before or during pandemic (χ^2^_(3)_ = 3.09; *p* = .38).

In the COVID-19 groups (LPR vs HRP) no woman resulted positive to virus and have had contact with person COVID-19 positive.

### Difference in EPDS score

Table 3 reported the difference among groups for EPDS scores.

**Table 3:** Comparison between groups for clinical data of Edinburgh Postnatal Depression Scale (EPDS)

	Pre- COVID-19				COVID-19			
	LRP		HRP		LRP		HRP	
	N	%	N	%	N	%	N	%
EPDS, mean (SD)	5.74 (4.91)		10.24 (6.04)		9.03 (4.98)		9.66 (4.47)	
Negative to depression (EPDS < 10)	32	82.1	17	45.9	22	59.5	18	47.4
Positive to depression (EPDS ≥10)	7	17.9	20	54.1	15	40.5	20	52.6

*LRP* Low-Risk Pregnancy, *HRP* High-Risk Pregnancy

The univariate analysis of Covariance ANCOVA carried out was significant for the main effects only of the clinical group [F (1,146) = 9.47.254, *p* = .008, η^2^ = .05]. Specifically, HRP group presented higher level of depressive symptoms according to EPDS compared to LRP group (M = 9.95; SD = 5.28 vs M = 7.34; SD = 5.19). On the contrary the ANCOVA was not significant for the main effects of COVID-19 group [F (1, 146) = 2.81, *p* = .096, η^2^ = .02] (pre-COVID-19 M = 7.93; SD = 5.90 vs COVID-19 M = 9.35; SD = 4.71). In fact, any difference merged in depressive symptom level between women pregnant before and during pandemic.

Finally, there was significant interaction effect for COVID-19 (before/ during pandemic) group X obstetric condition (LPR vs HRP [F (1,146) = 5.36, *p* = .022, η^2^ = .04]. Analysis of variance showed that in pre-COVID-19 group, hospitalized women with HRP presented higher level of depressive symptom than women with LRP [F (1,74) = 12.74, *p* = .001] while any difference exist in COVID-19 group between HRP and LRP [F (1,73) = .33, *p* = .565]. Moreover, the prevalence of participants with clinical depression according to EPDS was different in four groups (Chi square (3) = 13.296; *p* = .004) (Table 3).

The open-ended questions about the impact of COVID-19 and consequent restrictions on the emotional experience of pregnancy highlighted the main themes below reported. Quotations are reported indicating ID women (*#*Wn) who expressed. From the analysis of the open questions emerge some main themes in the two respective groups recruited during the COVID-19 period.

### The impact of pandemic restriction health services on pregnancy experience: practical and emotional aspects

#### Pregnancy, COVID-19 and isolation

More than half of LRP women express concerns due to the COVID-19 pandemic both for them and for their relatives. LRP showed fear of contagion for their older children (“*I’m afraid to send older children to school” #W4*) and of infecting others that lead them to enhance their isolation (“...*I’m afraid to see my parents for fear of getting infected …*” *#W10; “My husband took care of going out for the strictly necessary because I’m afraid to go to the supermarket so I still feel even more isolated” #W9).*

On the contrary HRP did not express concerns about COVID-19 in terms of personal infection and vertical transmission in the present moment. After the hospital admission, the women were tested for COVID-19 and while waiting for negative swab result, they experience a total isolation in COVID-19 bubble ward. During this permanence anxieties emerge from all women *(*i.e. *“I spent the first 24 hours in preventive isolation waiting for the swab responses I was afraid of being infected” #W2).* After the negative results of swab, the HRP were moved to COVID-free ward being undergone to frequent swab thus not reporting COVID-19 concerns about themselves.

#### Pregnancy visits: greater attention to medical aspects

Two third of LRP women reported a greater attention paid by medical staff to their own physical/clinical health and to the medical aspect of pregnancy respect to their psychological wellbeing as pregnant women. Women with LRP reported that routine control visits have been reduced, time-shifted not allowing to receive acceptable medical information. Also concerning labour and delivery most of LRP reported to not having the adequate medical information. Most of HRP express fears and concerns about foetus and the ongoing of pregnancy due to the reason of admission. With the admission to obstetric COVID-free ward and after few days one third of women recognized the importance of being hospitalized as a protective factor for mother and foetus health (“*Inside the hospital I feel safe for myself and my baby” #*W22). The information provided by medical professionals are frequent and let women having control about pregnancy. However they reported a lack of consideration of psychological aspect of hospitalization by medical staff.

#### The absence of partner during pregnancy routine visits and ultrasound

Despite great attention perceived about the physical health by medical staff, almost all women reported a lack of sensitivity about the consideration of pregnancy as a moment that needs to be shared with their partner. LRP women reported that their partner was not allowed during ultrasound examination and they expressed sadness and loneliness about not experiencing this moment of pregnancy together *(*i.e. *“My partner was unable to attend the visits and I was very sorry” #W2*). Almost all LRP would share the ultrasound experience with their partner (for instance making a movies of ultrasound) but it is not allowed (“*The partner is not allowed to participate and I was very sorry because he couldn’t see anything”#W2).* Also the childbirth is perceived by half of LRP as an event where the lack of partner is highlighted (“*thinking to face childbirth without him [partner], … has greatly de-stabilized me” #W7*).

Also almost all HRP reported the absence of partner as a source of discomfort *(*i.e. *“I need the physical presence of my son and husband” #W3).* Some women attempt to experience the pregnancy with their partner by means of daily phone calls but this contact is not enough to replace this need.

#### Family management and childcare during pandemic is source of stress

About one third of LRP has at least an older child, all these women reported a great burden related to the child management. Pandemic social restriction reduce occasions of socialization for children, reduce sport and leisure activities and, in some cases, the school attendance in presence. With their kids at home for on-line lesson women reported a sense of overwhelmed about child management together with housework (i.e. “*… was very tiring because my first child not go to school and also have to take care of houseworking..”#W12).* This burden is increased by the almost total absence of aid by grandparents who, constituting a high risk population for infection, are preserved by taking care of children.

Analogue concerns are expressed by most of HRP women who, not allowed to manage older children and housework due to their risky condition, tend to self-blame and express worries. HRP are aware that in pandemic condition, the home care and child care should be almost entirely managed by parents and they reported a sense of overwhelmed for not being able to contribute to family management due to the hospitalization despite their wish *(“I would like to take care of my daughter every day” #*W24). HRP women express the difficulties delegating the management of the home and care of the children to their partner (i.e.*“my greatest worry is that my husband is in trouble managing others child and housework in addition to his work.” #W11*). Almost all of HRP are afraid about the partner may be less supported in managing the other children ( *“… my husband would get help from his mother but she is old and afraid of contracting the COVID infection..” #W6).*

#### The social comparison and support as a protective factor

LRP women reported the lack of occasions to share their experience. One fifth of LRP reported that *“lack a space where to share the personal experience of pregnancy” (#W 15)*. The lack of adequate services reported by LRP is translated in the *“lack of a mental space to reflect about motherhood and child to be” (#W 10)*.

On the contrary, more than half of HRP reported feelings of support in the relationship with other roommates or other hospitalized women. Sharing similar clinical histories gives them the courage to experience this particular clinical condition (i.e. “… *the other girls hospitalized were very helpful, we also have a lot of laughs and this helps to distract us …” #W25; “..the girl in the room with me is moral support and I am for her. In this sense we feel lucky..” #W27; “… with some hospitalized women we all meet together at a set time ... it’s nice to be able to share our story makes us feel less alone” #W28).*

## Discussion

The COVID-19 restrictions represent a significant risk factor for perinatal mental health. To our knowledge, no study analyzed the impact of both COVID-19 condition (antecedent and during pandemic) and obstetric condition (LRP vs HRP) on the depressive symptoms in pregnant women. Our results underlined significant interaction effect between obstetric condition (LPR vs HRP) and COVID-19 condition. In fact, while in the pre-COVID-19 period, HRP presented higher score for depressive symptoms (and over the clinical cut-off) than LRP in line with previous literature [12–14], during the COVID-19 period, the two groups (HRP and LRP) did not significantly differ in depressive symptoms being the EPDS score close to cut-off for both groups. Therefore COVID-19 restrictions seemed to have a negative effect on the depressive symptoms of LRP women in a greater measure than HRP. On the contrary HRP, reporting similar EPDS scores both in pre- and during COVID-19 pandemic, seemed to be less affected by pandemic condition. In line with the previous studies we can hypothesize that the own medical issues reported by HRP women would take precedence over the stress of the pandemic; itcould explain the reason why there are no differences between the pre- and during- COVID-19 in HRP [24].

Our qualitative results help to clarify the impact of COVID-19 restrictions on life, emotions and thoughts of pregnant women with LRP and HRP.

For instance, the fear of COVID-19 contagion and its vertical transmission appeared as a common theme in LRP but not in the HRP. Being hospitalized and regularly screened for COVID-19 infection, could lead HRP group to minimize the concerns about COVID-19 focusing on their obstetric risk condition. On the contrary the LRP perceive themselves at risk of contagion and expressed fear of becoming infected or transmitting the virus both in a vertical line and to relatives in line with the previous study [25]. This fear led LRP women to limit their occasion of social life (i.e. remaining at home, delegating the shopping to partners) thus increasing social isolation, and a sense of loneliness with an increased risk of depressive symptoms [25].

Secondly despite both groups (HRP and LRP) reported a greater attention by clinical staff to the medical aspect of pregnancy HRP and LRP reported different experiences. HRP, conscious of their high-risk condition, expressed a sense of protection correlated to the hospitalization, for them and their baby. On the contrary LRP reported a paucity of attention to their pregnancy experience by medical staff who seems to be focused only on clinical management neglecting their emotional needs.

This paucity of attention to the pregnancy experience increased the dissatisfaction among LRP who seemed to consider negligent the level of care received [26].

Additionally, LPR reported a scarcity of social exchange with other pregnant women with whom they could have shared emotions and concerns. Therefore due to this paucity of social exchange LRP reported a great sense of loneliness. Conversely, HRP reported a sense of community and a perceived social support by roommates who, sharing the same clinical and obstetric condition, may help them to explore the emotional experience of pregnancy, and feelings also linked to the high-risk condition preventing possible depressive symptoms [2] in line with previous study [7].

Pregnant women during COVID-19 were afraid about the perceived lack of support by family members during pregnancy and they expressed concerns whether their relatives would be present during the perinatal period because of quarantine measures [27]. Despite the unchanged presence of a partner in the domestic context, and the possibility to share the pregnancy progress (perception of foetal movements, seeing the belly growing), the absence of partner during obstetric visits may reduce the awareness about the child-to-be. Thus LRP women may perceive a great loneliness and sadness due to the different experience of pregnancy compared to their partner. The obstetric visits are not shared within couple because partner is not admitted. Consequently partner cannot: be reassured about the progression of pregnancy; ask questions; be informed about following pregnancy steps; see the fetal image in ultrasound. This loneliness perceived by LRP during COVID-19 may constitute an additional risk factor for the increase of depressive symptoms [28] compared to pre-COVID-19 period. The HRP also reported the lack of partners as a critical point. Despite reporting the social support of other hospitalized women HRP expressed concern about the lack of physical presence of the partner perceived by the hospital institutions as visitors instead of as a primary source of physical and emotional support [29]*.*

For both groups of women, having older children appears to be an additional burden due to the additional responsibilities in caring for children during the pandemic restrictions.

In line with the Italian context where a great role disparity exists over time dedicated to household (being men less involved) [30], LRP reported a sense of weariness due to the management of older children together with housework. This weareness may constitute an additional risk factor for depressive symptoms [31, 32] also in our sample.

On the contrary HRP reported self-blame not being able to manage older children and housework due to their absence at home confirming the role disparity typical of the Italian context [33] and the difficulty in delegating these tasks to their partner.

Overall this study offered a more detailed picture about the impact of COVID-19 restrictions on depressive symptoms in LPR and HRP deepening the amount of emotions and concerns about their becoming mothers during the pandemic.

### Limitations

Despite the relevance of this study, it does present some limitations. Compared to a previous research on the impact of COVID-19 [34] this study presented a small sample size and low statistical power. However it must be noted that our sample is homogeneous for clinical, demographic and institutional characteristics being recruited in the same hospital setting in the pre-COVID-19 and during COVID-19. This homogeneity appears to be relevant because pregnant women of our sample (both high and low risk) received analogous clinical assistance in pre- and during COVID-19 period except for the restrictions due to the pandemic. Secondly, the HRP was selected using only the condition of risk for premature delivery neglecting other conditions such as maternal pathology. Despite this the relative homogeneity of this sample allows to generalize the results of this specific obstetric population.

Thirdly, we used only one standardized questionnaire (EPDS) to assess depressive symptoms. Although it could be interesting insert other questionnaires to assess other aspects of maternal mental health, we consider that also using a qualitative method could overcome this limit.

Regarding the focus on two groups during the COVID-19 restrictions, it must be remarked that the data of HRP women were collected in a COVID free ward after a confirmation from negative swabs. The certainty to be negative, together with the awareness to have no relatives tested positive may explain why the HRP did not report fear of infection. Future studies could investigate the levels of depressive symptoms in HRP comparing those in a COVID ward and in COVID free ward. In fact, the social network with other patients reported by HRP of our sample may not be present in the HRP women admitted to a COVID ward. It is thus possible that in a COVID ward HRP women may experience a greater sense of loneliness and isolation, as well as a higher concern about COVID-19 infection compared to those in COVID free ward with a more negative impact on depressive symptoms. This aspect should be clarified by future studies.

Also LRP resulted not infected by the virus and to their knowledge they had no direct contact with the virus. It may be possible that LRP women with COVID-19 positive swab or with direct contact with person affected by COVID-19 may report different worries. For this reason it could be desirable to make further investigations considering the COVID-19 infection or the presence of affected family members.

## Conclusions

In conclusion the COVID-19 restrictions increases depressive symptoms more in LPR than HRP compared to the previous period. In a context of strict restriction due to pandemic, the HRP seem to use hospitalization as a resource to find a social network support from other pregnant women and to be reassured on the clinical ongoing of pregnancy.

Despite the previous limitations this study constitutes an innovative point of view on the impact of pandemic restrictions on depressive symptoms and concerns of LRP and HRP women. A careful analysis of these risk factors could limit their negative effects on the well-being of this target population.

## Data Availability

the datasets used and/or analysed during the current study are available from the corresponding author on reasonable request.

## Abbreviations

LRP: Low-Risk Pregnancy
HRP: High-Risk Pregnancy
EPDS: Edinburgh Postnatal Depression Scale

## References

[1] Ayaz R, Hocaoğlu M, Günay T, Yardımcı OD, Turgut A, Karateke A (2020). Anxiety and depression symptoms in the same pregnant women before and during the COVID-19 pandemic. JPM.

[2] Ceulemans M, Hompes T, Foulon V (2020). Mental health status of pregnant and breastfeeding women during the COVID-19 pandemic: a call for action. IJGO.

[3] Durankuş F, Aksu E (2020). Effects of the COVID-19 pandemic on anxiety and depressive symptoms in pregnant women: a preliminary study. J Matern Fetal Neonatal Med.

[4] Lebel C, MacKinnon A, Bagshawe M, Tomfohr-Madsen L, Giesbrecht G (2020). Elevated depression and anxiety symptoms among pregnant individuals during the COVID-19 pandemic. JAD.

[5] Molgora S, Accordini M (2020). Motherhood in the time of coronavirus: the impact of the pandemic emergency on expectant and postpartum Women's psychological well-being. Front Psychol.

[6] Delmastro M, Zamariola G (2020). Depressive symptoms in response to COVID-19 and lockdown: across-sectional study on the Italian population. Sci Rep.

[7] Ostacoli L, Cosma S, Bevilacqua F, Berchialla P, Bovetti M, Carosso AR (2020). Psychosocial factors associated with postpartum psychological distress during the Covid-19 pandemic: a cross-sectional study. BMC Pregnancy Childbirth.

[8] Zanardo V, Manghina V, Giliberti L, Vettore M, Severino L, Straface G (2020). Psychological impact of COVID-19 quarantine measures in northeastern Italy on mothers in the immediate postpartum period. Int J Gynecol Obstet.

[9] Ravaldi C, Wilson A, Ricca V, Homer C, Vannacci A (2020). Pregnant women voice their concerns and birth expectations during the COVID-19 pandemic in Italy. Women Birth.

[10] Grote NK, Bridge JA, Gavin AR, Melville JL, Iyengar S, Katon WJ (2010). A meta-analysis of depression during pregnancy and the risk of preterm birth, low birth weight, and intrauterine growth restriction. Arch Gen Psychiatry.

[11] World Health Organization (2012). Born too soon: the global action report on preterm birth.

[12] Thiagayson P, Krishnaswamy G, Lim ML, Sung SC, Haley CL, Fung DSS (2013). Depression and anxiety in Singaporean high-risk pregnancies—prevalence and screening. Gen Hosp Psychiat.

[13] Pisoni C, Garofoli F, Tzialla C, Orcesi S, Spinillo A, Politi P (2016). Complexity of parental prenatal attachment during pregnancy at risk for preterm delivery. J Matern Fetal Neonatal Med.

[14] Dagklis T, Tsakiridis I, Chouliara F, Mamopoulos A, Rousso D, Athanasiadis A, Papazisis G (2018). Antenatal depression among women hospitalized due to threatened preterm labor in a high-risk pregnancy unit in Greece. J Matern Fetal Neonatal Med.

[15] Smorti M, Ginobbi F, Simoncini T, Pancetti F, Carducci A, Mauri G, et al. Anxiety and depression in hospitalized women due to high-risk pregnancy: an integrative quantitative and qualitative study. Curr Psychol. 2021. 10.1007/s12144-021-01902-5.

[16] Bateman BT, Mhyre JM, Hernandez-Diaz S, Huybrechts KF, Fischer MA, Creanga AA, et al. Development of a comorbidity index for use in obstetric patients. Obstet Gynecol. 2013;122(5). 10.1097/AOG.0b013e3182a603bb. 10.1097/AOG.0b013e3182a603bb PMC3829199 24104771

[17] Martin JA, Osterman MJK (2018). Describing the increase in preterm births in the United States, 2014-2016. NCHS Data Brief.

[18] Tucker J, McGuire W (2004). Epidemiology of preterm birth. BMJ.

[19] Cox JL, Holden JM, Sagovsky R (1987). Detection of postnatal depression. Development of the 10-item Edinburgh postnatal depression scale. Br J Psychiatry.

[20] Benvenuti P, Ferrara M, Niccolai C, Valoriani V, Cox JL (1999). The Edimburgh postnatal depression scale: validation for an Italian sample. JAD.

[21] Kozinszky Z, Dudas RB (2015). Validation studies of the Edinburgh postnatal depression scale for the antenatal period. J Affect Disord.

[22] Bowling A. Research methods in health: investigating health and health services. Maidenhead: McGraw-hill education (UK); 2014.

[23] Forrest Keenan K, van Teijlingen E, Pitchforth E (2005). The analysis of qualitative research data in family planning and reproductive health care. JFPRHC.

[24] Sade S, Sheiner E, Wainstock T, Hermon N, Yaniv Salem S, Kosef T (2020). Risk for depressive symptoms among hospitalized women in high-risk pregnancy units during the COVID-19 pandemic. J Clin Med.

[25] Serafini G, Parmigiani B, Amerio A, Aguglia A, Sher L, Amore M (2020). The psychological impact of COVID-19 on the mental health in the general population. QJM.

[26] Dodgson JE, Tarrant M, Chee YO, Watkins A (2010). New mothers’ experiences of social disruption and isolation during the severe acute respiratory syndrome outbreak in Hong Kong. Nurs Health Sci.

[27] Rashidi Fakari F, Simbar M (2020). Coronavirus pandemic and worries during pregnancy; a letter to editor. AAEM.

[28] Field T (2017). Prenatal depression risk factors, developmental effects and interventions: a review. J Preg Child Health.

[29] Ravaldi C, Mosconi L, Crescioli G, Ricca V, Vannacci A (2020). Are pregnant women satisfied with perinatal standards of care during COVID-19 pandemic? medRxiv.

[30] ISTAT (2019). I tempi della vita quotidiana lavoro, conciliazione, parità di genere e benessere soggettivo.

[31] Gawlik S, Müller M, Hoffmann L, Dienes A, Wallwiener M, Sohn C (2014). Prevalence of paternal perinatal depressiveness and its link to partnership satisfaction and birth concerns. Arch Wom Ment Health.

[32] Molgora S, Fenaroli V, Malgaroli M, Saita E (2017). Trajectories of postpartum depression in Italian first-time fathers. AJMH.

[33] Campolo MG, Di Pino A, Rizzi EL (2020). The labour division of Italian couples after a birth: assessing the effect of unobserved heterogeneity. J Popul Res.

[34] Ravaldi C, Ricca V, Wilson A, Homer C, Vannacci A (2020). Previous psychopathology predicted severe COVID-19 concern, anxiety, and PTSD symptoms in pregnant women during “lockdown” in Italy. Arch Wom Ment Health..

